# Supplementary material for: Endophytic Colletotrichum Species from Aquatic Plants in Southwest China
Source: J Fungi (Basel). 2022 Jan 16;8(1):87. doi: 10.3390/jof8010087 (PMC8779291; doi:10.3390/jof8010087)
Supplement: Supplementary file 1 [file jof-08-00087-s001.zip › jof-1546437-supplementary.pdf]

Table S1. GenBank accession numbers of taxa used in phylogenetic analyses.

Species name	Isolate No.	GenBank Accession No.				
		ITS	GAPDH	CHS-1	ACT	TUB2
Colletotrichum abscissum	COAD 1877	KP843126	KP843129	KP843132	KP843141	KP843135
Colletotrichum acutatum	CBS 112996	JQ005776	JQ948677	JQ005797	JQ005839	JQ005860
Colletotrichum aenigma	ICMP 18608	JX010244	JX010044	JX009774	JX009443	JX010389
Colletotrichum aeschynomenes	ICMP 17673	JX010176	JX009930	JX009799	JX009483	JX010392
Colletotrichum agaves	CBS 118190	DQ286221	-	-	-	-
Colletotrichum alatae	CBS 304.67	JX010190	JX009990	JX009837	JX009471	JX010383
Colletotrichum alienum	ICMP 12071	JX010251	JX010028	JX009882	JX009572	JX010411
Colletotrichum annellatum	CBS 129826	JQ005222	JQ005309	JQ005396	JQ005570	JQ005656
Colletotrichum aotearoa	ICMP 18537	JX010205	JX010005	JX009853	JX009854	JX010420
Colletotrichum arecicola	CGMCC 3.19667	MK914635	MK945455	MK935541	MK935374	MK935498
Colletotrichum artocarpicola	MFLUCC 18-1167	MN415991	MN435568	MN435569	MN435570	MN435567
Colletotrichum asianum	ICMP 18580	JX010196	JX010053	JX009867	JX009584	JX010406
Colletotrichum baiyuense	BY64	OL842182	OL981277	OL981303	OL981251	-
Colletotrichum baiyuense	BY75	OL842183	OL981278	OL981304	OL981252	-
Colletotrichum baiyuense	YMF 1.04941	OL842184	OL981279	OL981305	OL981253	-
Colletotrichum beeveri	CBS 128527	JQ005171	JQ005258	JQ005345	JQ005519	JQ005605
Colletotrichum boninense	CBS 123755	JQ005153	JQ005240	JQ005327	JQ005501	JQ005588
Colletotrichum brasiliense	CBS 128501	JQ005235	JQ005322	JQ005409	JQ005583	JQ005669
Colletotrichum brassicicola	CBS 101059	JQ005172	JQ005259	JQ005346	JQ005520	JQ005606
Colletotrichum brevisporum	BCC 38876	JN050238	JN050227	-	JN050216	JN050244
Colletotrichum cacao	CBS 119297	MG600772	MG600832	MG600878	MG600976	MG601039
Colletotrichum camelliae	CGMCC:3.14925	KJ955081	KJ954782	-	KJ954363	KJ955230
Colletotrichum camelliae-japonicae	CGMCC3.18118	KX853165	KX893584	-	KX893576	KX893580

Colletotrichum casaense	BY97	OL842178	OL981273	OL981299	OL981247	-
Colletotrichum casaense	KS17	OL842179	OL981274	OL981300	OL981248	-
Colletotrichum casaense	YMF 1.04947	OL842180	OL981275	OL981301	OL981249	-
Colletotrichum casaense	LT2	OL842181	OL981276	OL981302	OL981250	-
Colletotrichum catinaense	CBS 142417	KY856400	KY856224	KY856136	KY855971	KY856482
Colletotrichum cattleyicola	CBS 170.49	MG600758	MG600819	MG600866	MG600963	MG601025
Colletotrichum caudatum	CBS 131602	JX076860	-	-	-	-
Colletotrichum changpingense	MFLUCC 15-0022	KP683152	KP852469	KP852449	KP683093	KP852490
Colletotrichum chlorophyti	IMI 103806	GU227894	GU228286	GU228384	GU227992	GU228188
Colletotrichum chrysophillum	CMM4268	KX094252	KX094183	KX094083	KX093982	KX094285
Colletotrichum ciggaro	ICMP 18539	JX010230	JX009966	JX009800	JX009523	JX010434
Colletotrichum citricola	CBS 134228	KC293576	KC293736	KC293792	KC293616	KC293656
Colletotrichum citrus-medicae	GUCC 1554	MN959910	MT006331	MT006328	MT006325	-
Colletotrichum clidemiae	ICMP 18658	JX010265	JX009989	JX009877	JX009537	JX010438
Colletotrichum cliviicola	CBS 125375	MG600733	MG600795	MG600850	MG600939	MG601000
Colletotrichum cobbittiense	BRIP 66219a	MH087016	MH094133	MH094135	MH094134	MH094137
Colletotrichum coccodes	CBS 369.75	HM171679	HM171673	JQ005796	HM171667	JQ005859
Colletotrichum colombiense	CBS 129818	JQ005174	JQ005261	JQ005348	JQ005522	JQ005608
Colletotrichum condaoense	CBS 134299	MH229914	MH229920	MH229926	-	MH229923
Colletotrichum conoides	CAUG17*	KP890168	KP890162	KP890156	KP890144	KP890174
Colletotrichum constrictum	CBS 128504	JQ005238	JQ005325	JQ005412	JQ005586	JQ005672
Colletotrichum cordylinicola	ICMP 18579	JX010226	JX009975	JX009864	HM470234	JX010440
Colletotrichum costaricense	CBS 330.75	JQ948180	JQ948510	JQ948841	JQ949501	JQ949831
Colletotrichum curcumae	IMI 288937	GU227893	GU228285	GU228383	GU227991	GU228187
Colletotrichum cuscutae	IMI 304802	JQ948195	JQ948525	JQ948856	JQ949516	JQ949846
Colletotrichum cymbidiicola	IMI 347923	JQ005166	JQ005253	JQ005340	JQ005514	JQ005600

Colletotrichum dacrycarpi	CBS 130241	JQ005236	JQ005323	JQ005410	JQ005584	JQ005670
Colletotrichum dematium	CBS 125.25	GU227819	GU228211	GU228309	GU227917	GU228113
Colletotrichum demersi	HF18	OL842176	OL981271	OL981297	OL981245	OL981231
Colletotrichum demersi	YMF 1.04946	OL842177	OL981272	OL981298	OL981246	OL981232
Colletotrichum destructivum	CBS 136228	KM105207	KM105561	KM105277	KM105417	KM105487
Colletotrichum dianense	YMF 1.04943	OL842189	OL981284	OL981310	OL981258	-
Colletotrichum doitungense	MFLUCC 14-0128	MF448524	MH049480	-	MH376385	MH351277
Colletotrichum dracaenophilum	CBS 118199	JX519222	JX546707	JX519230	JX519238	JX519247
Colletotrichum endophytica	MFLUCC 13–0418	KC633854	KC832854	-	KF306258	-
Colletotrichum eryngiicola	MFLUCC 17-0318	KY792726	KY792723	KY792720	KY792717	KY792729
Colletotrichum euphorbiae	CBS 134725	KF777146	KF77713	KF777128	KF777125	KF777247
Colletotrichum feijoicola	CBS 144633	MK876413	MK876475	-	MK876466	MK876507
Colletotrichum fioriniae	CBS 128517	JQ948292	JQ948622	JQ948953	JQ949613	JQ949943
Colletotrichum fructicola	ICMP 18581	JX010165	JX010033	JX009866	FJ907426	JX010405
Colletotrichum fructicola	YMF 1.04959	OL842164	OL981259	OL981285	OL981233	-
Colletotrichum fructicola	Y79-6	OL842165	OL981260	OL981286	OL981234	-
Colletotrichum fructivorum	CBS 133125	JX145145	-	-	-	JX145196
Colletotrichum gigasporum	CBS 133266	KF687715	KF687822	KF687761	-	KF687866
Colletotrichum gloeosporioides	CBS 112999	JQ005152	JQ005239	JQ005326	JQ005500	JQ005587
Colletotrichum graminicola	CBS 130836	JQ005767	-	JQ005788	JQ005830	JQ005851
Colletotrichum grevilleae	CBS 132879	KC297078	KC297010	KC296987	KC296941	KC297102
Colletotrichum grossum	CAUG7*	KP890165	KP890159	KP890153	KP890141	KP890171
Colletotrichum guizhouensis	CGMCC 3.15112	JX625158	KC843536	-	KC843536	JX625185
Colletotrichum hebeiense	MFLUCC13–0726	KF156863	KF377495	KF289008	KF377532	KF288975
Colletotrichum hederiicola	MFLU 15-0689	MN631384	-	MN635794	MN635795	-
Colletotrichum helleniense	CBS 142418	KY856446	KY856270	KY856186	KY856019	KY856528

Colletotrichum henanense	CGMCC 3.17354	KJ955109	KJ954810	-	KM023257	KJ955257
Colletotrichum hippeastri	CBS 125376	JQ005231	JQ005318	JQ005405	JQ005579	JQ005665
Colletotrichum horii	ICMP 10492	GQ329690	GQ329681	JX009752	JX009438	JX010450
Colletotrichum hsienjenchang	MAFF 243051	AB738855	-	AB738846	AB738845	-
Colletotrichum hystricis	CBS 142411	KY856450	KY856274	KY856190	KY856023	KY856532
Colletotrichum jasminigenum	MFLUCC 10-0273	HM131513	-	-	-	HM153770
Colletotrichum jiangxiense	CGMCC 3.17363	KJ955201	KJ954902	-	KJ954471	KJ955348
Colletotrichum jiangxiense	YMF 1.04949	OL842166	OL981261	OL981287	OL981235	-
Colletotrichum jiangxiense	QC67	OL842167	OL981262	OL981288	OL981236	-
Colletotrichum jiangxiense	HM6	OL842168	OL981263	OL981289	OL981237	-
Colletotrichum kahawae	ICMP17816	JX010231	JX010012	JX009813	JX009452	JX010444
Colletotrichum karstii	CORCG6	HM585409	HM585391	HM582023	HM581995	HM585428
Colletotrichum karstii	YMF 1.04944	OL842174	OL981269	OL981295	OL981243	OL981229
Colletotrichum liaoningense	CGMCC 3.17616	KP890104	KP890135	KP890127	KP890097	KP890111
Colletotrichum lilii	CBS 109214	GU227810	GU228202	GU228300	GU227908	GU228104
Colletotrichum limetticola	CBS 114.14	JQ948193	JQ948523	JQ948854	JQ949514	JQ949844
Colletotrichum limonicola	CBS 142410	KY856472	KY856296	KY856213	KY856045	KY856554
Colletotrichum litangense	YMF 1.04948	OL842185	OL981280	OL981306	OL981254	-
Colletotrichum litangense	LT31	OL842186	OL981281	OL981307	OL981255	-
Colletotrichum litangense	LT76	OL842187	OL981282	OL981308	OL981256	-
Colletotrichum lobatum	IMI 79736	MG600768	MG600828	MG600874	MG600972	MG601035
Colletotrichum lupine	CBS 109225	JQ948155	JQ948485	JQ948816	JQ949476	JQ949806
Colletotrichum magnum	CBS 519.97	MG600769	MG600829	MG600875	MG600973	MG601036
Colletotrichum makassarense	CBS 143664a,	MH728812	MH728820	MH805850	MH781480	MH846563
Colletotrichum melonis	CBS 159.84	JQ948194	JQ948524	JQ948855	JQ949515	JQ949845
Colletotrichum merremiae	CBS 124955	MG600765	MG600825	MG600872	MG600969	MG601032

Colletotrichum metake	MAFF 244029	AB738859	-	-	-	-
Colletotrichum musae	ICMP19119	HQ596292	HQ596299	JX009896	HQ596284	HQ596280
Colletotrichum musicola	CBS 132885	MG600736	MG600798	MG600853	MG600942	MG601003
Colletotrichum navitas	CBS 125086	JQ005769	-	JQ005790	JQ005832	JQ005853
Colletotrichum nigrum	CBS 169.49	JX546838	JX546742	JX546693	JX546646	JX546885
Colletotrichum novae-zelandiae	CBS 128505	JQ005228	JQ005315	JQ005402	JQ005576	JQ005662
Colletotrichum nupharicola	ICMP 18187	JX010187	JX009972	JX009835	JX009437	JX010398
Colletotrichum ocimi	CBS 298.94	KM105222	KM105577	KM105292	KM105432	KM105502
Colletotrichum okinawense	MAFF 240517	MG600767	MG600827	-	MG600971	MG601034
Colletotrichum oncidii	CBS 129828	JQ005169	JQ005256	JQ005343	JQ005517	JQ005603
Colletotrichum orbiculare	CBS 570.97	KF178466	KF178490	KF178515	KF178563	KF178587
Colletotrichum orchidearum	CBS 135131	MG600738	MG600800	MG600855	MG600944	MG601005
Colletotrichum orchidis	MFLUCC17-1302	MK502144	MK496857	MK496855	MK496853	MK496859
Colletotrichum orchidophilum	CBS 632.80	JQ948151	JQ948481	JQ948812	JQ949472	JQ949802
Colletotrichum panamense	CBS 125386	MG600766	MG600826	MG600873	MG600970	MG601033
Colletotrichum pandanicola	MFLUCC 17-0571	MG646967	MG646934	MG646931	MG646938	MG646926
Colletotrichum paranaense	CBS 134729	KC204992	KC205026	KC205043	KC205077	KC205060
Colletotrichum parsonsiae	CBS 128525	JQ005233	JQ005320	JQ005407	JQ005581	JQ005667
Colletotrichum perseae	CBS 141365	KX620308	KX620242	-	KX620145	KX620341
Colletotrichum petchii	CBS 378.94	JQ005223	JQ005310	JQ005397	JQ005571	JQ005657
Colletotrichum phaseolorum	CBS 158.36	GU227897	GU228289	GU228387	GU227995	GU228191
Colletotrichum philoxeroidis	YMF 1.04945	OL842188	OL981283	OL981309	OL981257	-
Colletotrichum phyllanthi	CBS 175.67	JQ005221	JQ005308	JQ005395	JQ005569	JQ005655
Colletotrichum piperis	IMI 71397	MG600760	MG600820	MG600867	MG600964	MG601027
Colletotrichum plurivorum	CBS 125474	MG600718	MG600781	MG600841	MG600925	MG600985
Colletotrichum proteae	CBS 132882	KC297079	KC297009	KC296986	KC296940	KC297101

Colletotrichum pseudoacutatum	CBS 436.77	JQ948480	JQ948811	JQ949141	JQ949801	JQ950131
Colletotrichum pseudomajus	CBS 571.88	KF687722	KF687826	KF687779	KF687801	KF687883
Colletotrichum pseudotheobromicola	MFLUCC 18–1602	MH817395	MH853675	MH853678	MH853681	MH853684
Colletotrichum psidii	CBS 145.29	JX010219	JX009967	JX009901	JX009515	JX010443
Colletotrichum pyrifolia	CGMCC 3.18902	MG748078	MG747996	MG747914	MG747768	MG748158
Colletotrichum queenslandicum	ICMP 1778	JX010276	JX009934	JX009899	JX009447	JX010414
Colletotrichum radicis	CBS 529.93	KF687719	KF687825	KF687762	KF687785	KF687869
Colletotrichum rhexiae	CBS 133134	JX145128	-	-	-	JX145179
Colletotrichum rusci	CBS 119206	GU227818	GU228210	GU228308	GU227916	GU228112
Colletotrichum salsolae	ICMP 19051	JX010242	JX009916	JX009863	JX009562	JX010403
Colletotrichum siamense	ICMP 18578	FJ972613	FJ972575	JX009865	FJ907423	FJ907438
Colletotrichum sidae	CBS 504.97	KF178472	KF178497	KF178521	KF178569	KF178593
Colletotrichum sojae	ATCC 62257	MG600749	MG600810	MG600860	MG600954	MG601016
Colletotrichum somersetense	CBS 131599	JX076862	-	-	-	-
Colletotrichum spaethianum	CBS 167.49	GU227807	GU228199	GU228297	GU227905	GU228101
Colletotrichum spicati	YMF 1.04942	OL842171	OL981266	OL981292	OL981240	OL981226
Colletotrichum spicati	F6	OL842172	OL981267	OL981293	OL981241	OL981227
Colletotrichum spinosum	CBS 515.97	KF178474	KF178498	KF178523	KF178571	KF178595
Colletotrichum sydowii	CBS135819	KY263783	KY263785	KY263787	KY263791	KY263793
Colletotrichum syzygiicola	MFLUCC 10-0624	KF242094	KF242156	-	KF157801	KF254880
Colletotrichum tainanense	CBS 143666a	MH728818	MH728823	MH805845	MH781475	MH846558
Colletotrichum tamarilloi	CBS 129814	JQ948184	JQ948514	JQ948845	JQ949505	JQ949835
Colletotrichum temperatum	CBS 133122	JX145159	-	-	-	JX145211
Colletotrichum tengchongense	YMF 1.04950	OL842169	OL981264	OL981290	OL981238	-
Colletotrichum theobromicola	ICMP 18649	JX010294	JX010006	JX009869	JX009444	JX010447
Colletotrichum ti	ICMP 4832	JX010269	JX009952	JX009898	JX009520	JX010442

Colletotrichum torulosum	CBS 128544	JQ005164	JQ005251	JQ005338	JQ005512	JQ005598
Colletotrichum trichellum	CBS 217.64	GU227812	GU228204	GU228302	GU227910	GU228106
Colletotrichum tropicale	CBS 124949	JX010264	JX010007	JX009870	JX009489	JX010407
Colletotrichum truncatum	CBS 151.35	GU227862	GU228254	GU228352	GU227960	GU228156
Colletotrichum viniferum	GZAAS5.08601	JN412804	JN412798	-	JN412795	JN412813
Colletotrichum vittalense	CBS 181.82	MG600734	MG600796	MG600851	MG600940	MG601001
Colletotrichum vulgaris	YMF 1.04940	OL842170	OL981265	OL981291	OL981239	-
Colletotrichum watphraense	MFLUCC 14-0123	MF448523	MH049479	-	MH376384	MH351276
Colletotrichum wuxiense	CGMCC 3.17894	KU251591	KU252045	KU251939	KU251672	KU252200
Colletotrichum wuxuhaiense	YMF 1.04951	OL842173	OL981268	OL981294	OL981242	OL981228
Colletotrichum wuxuhaiense	F34	OL842175	OL981270	OL981296	OL981244	OL981230
Colletotrichum xanthorrhoeae	ICMP 17903	JX010261	JX009927	JX009823	JX009478	JX010448
Colletotrichum yulongense	CFCC 50818	MH751507	MK108986	MH793605	MH777394	MK108987
Colletotrichum yunnanense	CBS 13213	JX546804	JX546706	JX519231	JX519239	JX519248
Monilochaetes infuscans	CBS 86996	JQ005780	JX546612	JQ005801	-	JQ005864
